# Addressing the Crimean-Congo Hemorrhagic Fever Crisis in Pakistan's Healthcare System: An Urgent Call for Holistic Action

**DOI:** 10.7759/cureus.56831

**Published:** 2024-03-24

**Authors:** Harendra Kumar, Arkadeep Dhali, Jyotirmoy Biswas, Gopal Krishna Dhali

**Affiliations:** 1 General Medicine, Dow University of Health Sciences, Karachi, PAK; 2 Gastroenterology, University of Sheffield, Sheffield, GBR; 3 Internal Medicine, Sheffield Teaching Hospitals NHS Foundation Trust, Sheffield, GBR; 4 General Medicine, College of Medicine and Sagore Dutta Hospital, Kolkata, IND; 5 Gastroenterology, School of Digestive and Liver Diseases, Institute of Postgraduate Medical Education and Research, Kolkata, IND

**Keywords:** outcome, public health, outbreak, pakistan, crimean-congo hemorrhagic fever virus

## Abstract

The recent resurgence of the Crimean-Congo hemorrhagic fever (CCHF) in Pakistan's Balochistan province has significantly impacted both the medical community and the general population. Initially perceived as a concerning development, the situation has deteriorated, culminating in the infection and mortality of healthcare workers directly engaged in managing this virulent outbreak. This critical situation necessitates an urgent and collective response, transcending national boundaries to involve the international healthcare community.

## Editorial

The Crimean-Congo hemorrhagic fever (CCHF), a viral hemorrhagic fever primarily vectored through ticks and potentially through contact with infected animal tissues, has demonstrated a propensity for instigating epidemics, notably within healthcare facilities across Africa, the Balkans, the Middle East, and certain regions of Asia [1,2]. The recent surge in CCHF incidents among healthcare professionals in Balochistan intensifies the disquietude surrounding the virus [3].

Balochistan's plight has rapidly worsened since August, marked by escalating hospitalizations in Quetta, the provincial capital. By mid-October, the tally stood at 41 confirmed cases, with a disheartening 15 fatalities [1]. The gravity of the situation compounded with the revelation on November 4, indicating an additional eight cases, five of which purportedly involved healthcare workers [1]. The subsequent issuance of a red alert by the Balochistan government in response to the 16 CCHF-related fatalities, including that of a doctor, serves as a stark reminder of the peril confronting those on the epidemic's frontlines [1]. The unwavering commitment of these healthcare stalwarts, risking their lives in the pursuit of caring for the afflicted, warrants our utmost veneration and unwavering support.

The genesis of a potential contagion among healthcare professionals following the admission of a CCHF-positive patient on October 22 emphasizes the precariousness of our healthcare infrastructure and accentuates the exigency for more efficacious infection control protocols [1-3]. The immediacy and comprehensiveness of our response are imperative in light of the criticality of the situation.

Pakistan's healthcare landscape, already grappling with a myriad of health challenges, now faces the compounding burden of a surge in CCHF cases in Balochistan [1,4]. The prevailing onslaught of infectious diseases, noncommunicable ailments, and maternal and child health issues accentuates the monumental strain on healthcare facilities and resources, perennially teetering on the brink of overextension [4].

In response to the escalating crisis, the government has instituted a series of measures encompassing the isolation of affected wards, the sanitization of cattle markets, and a temporary moratorium on private animal slaughter in public spaces for a fortnight [3]. While laudable and imperative, these measures constitute a mere prelude to the formidable struggle against CCHF [4,5]. Urgent actions are indispensable, yet concurrent long-term strategizing is imperative for the efficacious management of existing epidemics and the prophylaxis against future outbreaks. As the CCHF crisis unfolds, it is imperative to heed the strategic recommendations outlined for Pakistan's healthcare resilience (Table 1).

**Table 1:** Strategic recommendations and rationale for strengthening Pakistan's response to CCHF. CCHF: Crimean-Congo hemorrhagic fever

Recommendations	Rationale
Strengthening healthcare infrastructure	- A resilient healthcare infrastructure is paramount for effective crisis management.
	- Investments in facilities, resources, and skilled professionals ensure prompt and quality medical care.
	- Safeguarding both patients and healthcare workers is a crucial outcome of a robust infrastructure.
Protection and support for healthcare workers	- Prioritizing the safety and well-being of healthcare workers, the frontline heroes.
	- Providing comprehensive support, including appropriate protective gear, specialized training, and psychological assistance.
	- Recognizing and mitigating the challenges faced by healthcare professionals in their crucial roles.
Community engagement	- Engaging and educating communities is a powerful strategy for preventing infectious disease spread.
	- Informed communities are more likely to adopt preventive measures.
	- Utilizing community health workers, local leaders, and educational campaigns facilitates effective communication and awareness.
Rapid response teams	- The establishment of specialized rapid response teams is critical for agile outbreak management.
	- Comprising experts in infectious diseases, epidemiologists, and healthcare professionals.
	- Playing a pivotal role in swift identification, isolation, treatment, and contact tracing, preventing further transmission.
Research and vaccine development	- Investing in research is imperative to develop specific interventions for CCHF.
	- The absence of targeted treatments or vaccines underscores the need for robust research and development efforts.
	- Enhancing preparedness and response capabilities for future outbreaks is a crucial outcome of sustained research efforts.

The recent CCHF epidemic in Pakistan accentuates the imperative for a multifaceted approach to managing and preempting such health crises. A robust healthcare infrastructure, necessitating substantial investments to enhance institutions, furnish them with the requisite resources, and guarantee the availability of proficient healthcare personnel, emerges as an exigent mandate. The burgeoning CCHF caseload in Pakistan further stresses the exigency for fortifying healthcare facilities already grappling with overworked staff.

Simultaneously, safeguarding and supporting frontline healthcare workers assumes paramount significance, entailing the provision of not only protective equipment but also psychological succor. Community engagement, educational endeavors, and the formation of adept rapid response teams play pivotal roles in thwarting the dissemination of infectious diseases, mandating concerted collaboration with local leaders and healthcare professionals. Additionally, prioritizing research and vaccine development is imperative to augment our preparedness for impending CCHF outbreaks, considering the dearth of specific treatments or vaccines.

Amidst Pakistan's beleaguered healthcare system, contending with an array of health predicaments, collaborative endeavors emerge as the linchpin in efficaciously combating CCHF and addressing the overarching frailties of the healthcare infrastructure. The upswing in CCHF cases in Pakistan is an ominous quandary demanding ceaseless vigilance and commitment from all stakeholders. Despite the monumental challenge, it is not insurmountable. Implementation of the outlined recommendations, coupled with international collaboration, augurs well for augmenting our capacity to contend with CCHF outbreaks and fortifying the health and well-being of our populace.

Conclusion

This pandemic underscores the imperative for sustained investments in our healthcare system, the amplification of our public health infrastructure, and the dissemination of heightened public awareness and education. These measures are not merely reactive to existing challenges but proactive in fortifying our resilience against prospective health hazards. The collective battle against infectious diseases, particularly those with epidemic propensities, epitomizes a collaborative effort involving governments, healthcare institutions, researchers, and the broader public. It serves as an indelible reminder that investments in healthcare and public health are investments in our shared well-being and resilience.

## References

[1] (2023). Crimean-Congo hemorrhagic fever infects health workers in Pakistan outbreak. https://www.cidrap.umn.edu/viral-hemorrhagic-fever/crimean-congo-hemorrhagic-fever-infects-health-workers-pakistan-outbreak.

[2] Tabassum S, Naeem A, Khan MZ, Mumtaz N, Gill S, Ohadi L (2023). Crimean-Congo hemorrhagic fever outbreak in Pakistan, 2022: a warning bell amidst unprecedented floods and COVID 19 pandemic. Health Sci Rep.

[3] Kumar H, Bakhru D (2022). Rabies in Pakistan: a never ending challenge. Ann Med Surg (Lond).

[4] Tariq S, Niaz F, Safi Vahidy A, Qidwai M, Ishaq M, Abbasher Hussien Mohamed Ahmed K, Ullah I (2023). Crimean-Congo hemorrhagic fever (CCHF) in Pakistan: the daunting threat of an outbreak as Eid-ul-Azha approaches. Disaster Med Public Health Prep.

[5] 5 5 (2023). Crimean-Congo haemorrhagic fever. https://www.who.int/news-room/fact-sheets/detail/crimean-congo-haemorrhagic-fever.

